# One third of dementia cases can be prevented within the next 25 years by tackling risk factors. The case “for” and “against”

**DOI:** 10.1186/s13195-020-00646-x

**Published:** 2020-07-08

**Authors:** Manuel Montero-Odasso, Zahinoor Ismail, Gill Livingston

**Affiliations:** 1 Gait and Brain Lab, Parkwood Institute, Lawson Health Research Institute, London, ON Canada; 2 Schulich School of Medicine & Dentistry, Department of Medicine and Division of Geriatric Medicine, The University of Western Ontario, London, ON Canada; 3 Department of Epidemiology and Biostatistics, The University of Western Ontario, London, ON Canada; 4 Departments of Psychiatry, Clinical Neurosciences, and Community Health Sciences, Hotchkiss Brain Institute, Calgary, Canada; 5 O’Brien Institute for Public Health, University of Calgary, Calgary, Canada; 6 Division of Psychiatry, University College London, London, UK; 7 Camden and Islington NHS Foundation Trust, London, UK

**Keywords:** Debate, Dementia, Prevention, Lifestyle interventions, Multidomain trials

## Abstract

**Background:**

Recently, it has been suggested that up to a third of the dementia cases might be preventable. While prevention is always better than cure, this is particularly important in the field of dementia, as current interventions are not able to modify the disease. This article revises the evidence “for” and “against” dementia primary prevention.

**Discussion:**

Evidence “for” is sustained by the Lancet Commission on Dementia Prevention, Intervention and Care that noted a reduction of age-related incidence of dementia in high-income countries. Based on results from large cohort studies and using population attributable risk, the commission concluded that up to 35% of dementia cases could be prevented by modifying nine risk factors: low education, midlife hearing loss, obesity, hypertension, late-life depression, smoking, physical inactivity, diabetes, and social isolation. In this life course conceptual framework, modifications of risk factors can influence dementia decades before clinical disease onset. However, evidence “against” is supported by large randomized controlled trials (RCT, > 250 participants per arm, minimum of 6 months follow-up), primarily set to prevent dementia using lifestyle interventions that have shown modest or negative results. The 2017 National Academy of Medicine report concluded that the current evidence is limited and there are no specific interventions to warrant a public health recommendation for dementia prevention.

**Summary:**

Multiple pathological pathways are involved in the development of dementia which are theoretically treatable by managing midlife hearing loss and hypertension, and with physical exercise and education, as suggested by robust observational studies. However, evidence from large clinical trials is not conclusive to support that a third of dementia cases might be prevented. Current initiatives testing the effect of lifestyle interventions in larger clinical trials may help to settle this debate.

## Background

Over the next 30 years, the worldwide prevalence of Alzheimer’s disease (AD) and related dementias is expected to almost triple. Dementia carries enormous costs to the individual, their family, and to health and social care systems with global annual costs estimated to be more than US$800 billion [1]. Secondary prevention trials using pharmacological means have failed, mainly because our current understanding of the relationship and temporality between dementia pathology and impending cognitive impairment is incomplete. Multiple pathways are involved in the development of dementia including modifiable factors that can be categorized as (a) brain health in midlife (e.g., hypertension, obesity, smoking, physical activity), (b) cognitive ability and reserve (e.g., education), (c) performance in testing versus central damage (e.g., hearing loss), and (d) prodromal or reverse causation (e.g., depression, social isolation, physical inactivity) that are sometimes specific to different parts of the life course. Therefore, there has been a shift to primary prevention, and today, the focus is on treatment as prevention.

The Lancet Commission on Dementia Prevention, Intervention and Care Report [2] suggest that, if nine potentially reversible risk factors are considered, up to a third of the dementia cases might be preventable. While prevention is always better than cure, this is particularly important in the field of dementia as it takes years for the AD pathology to accumulate [3] and current interventions are not able to modify the disease once pathology is present.

For many years, we have hoped to be 5 to 10 years away from drugs that would modify dementia and AD to a clinically significant extent. Charities and clinicians have advocated and worked out the cost of organizing clinical pathways in expectation that everyone with high amyloid will be offered such drugs [4]. However, others have suggested that focusing on intervening for modifiable risk factors would be more effective [5].

During the 10th Canadian Conference on Dementia (CCD), in October 2019 in Québec City, we debated the arguments and evidence “for” and “against” whether up to a third of the dementia cases might be preventable within 25 years.

## Discussion

### Evidence supporting the affirmative view

“Be ambitious about prevention”

The Lancet Commission on Dementia [2].

At the time of this debate, there had been no positive results on cognition from RCTs to reduce brain amyloid load, with studies revealing negative results from monoclonal anti-Aβ antibodies, β-secretase 1 inhibitors (BACE1), and protease involved in the Aβ-peptides production. Similarly, anti-inflammatory drugs are so far disappointing [6]. Since then, the “Aducanumab trial”—which was stopped because of futility—has reported unpublished positive results in a subgroup of participants. Although positive findings in anti-Aβ antibody studies would be very welcome, the ethical implications of giving a drug with significant side effects to individuals with brain amyloid but no cognitive symptoms or brain atrophy, as most of whom would never develop AD [7], is still unresolved.

The Lancet Commission on Dementia noted a reduction of age-related incidence of dementia in several high-income countries, among those with more education or wealth. This suggests that it is possible to delay or prevent dementia. It concluded that up to 35% of dementia could be prevented by modifying nine risk factors: low education; midlife hearing loss, obesity, and hypertension; and late-life depression, smoking, physical inactivity, diabetes, and social isolation. An important aspect of this life course conceptual framework is that modifications of risk factors can be done by lifestyle interventions, which can influence dementia decades before clinical onset.

Assuming a 20% reduction in seven of the nine risk factors per decade, this would lead to a 15% reduction in dementia prevalence by the year 2050 [8]. However, key questions remain unanswered. For example, how, at which stage of the life course, and for how long any lifestyle interventions would need to be undertaken to address any of the risk factors are still unknown. There are also limitations when extrapolating observational findings to treatment effects mainly grounded in the unmeasured confounders. Similarly, reverse causation is an issue when using observational findings since social isolation, depression, and physical inactivity can certainly increase in frequency among those who are becoming cognitively impaired. Finally, although cardiovascular comorbidity at middle age is a risk factor for dementia, observational studies showed that vascular comorbidity in the oldest-old is no longer associated with incident dementia [8].

### Evidence supporting the opposing view

“It is a capital mistake to theorize before you have all the evidence. It biases the judgment.”

Arthur Conan Doyle

“A Study in Scarlett”, 1887

When evidence for lifestyle interventions is drawn from large randomized controlled trials (RCT, > 250 participants per arm, minimum of 6 months follow-up), primarily set to prevent dementia or progression to mild cognitive impairment, the results are modest or negative. Based on these trials, the 2017 National Academy of Medicine report concluded that the current evidence remains relatively limited and there are no specific interventions to warrant a public health recommendation for dementia prevention [9].

Among those large trials, three multidomain trials stand out: the Finnish Geriatric Intervention Study to Prevent Cognitive Impairment and Disability (FINGER) [10], the Multidomain Alzheimer Preventive Trial (MAPT) [11], and, the Dutch Prevention of Dementia by Intensive Vascular Care (PreDIVA) [12]. FINGER was a large, 2-year trial that established that combining a brain-healthy diet, exercise, cognitive training, and vascular risk monitoring helped to improve or maintain cognitive function in older adults at risk of AD. However, the control group that received usual care also showed cognitive improvements, and consequently, the effect size difference between control and intervention groups was very small, amounting to a Cohen’s *d* = 0.13. The unexpected significant improvement in the control group could also be due to learning effects of repeated cognitive testing [13], adding another layer of complexity to the interpretation of the results. Conversely, MAPT failed to find significant effects of multimodal lifestyle interventions, omega-3 supplementation, or a combination of the two on episodic memory. Considerations for this negative result include the older age and greater frailty in the MAPT participants, and the less rigorous delivery of the non-supervised physical exercise when compared with FINGER. Finally, the PreDIVA trial, a large multidomain cardiovascular intervention for dementia prevention, did not result in an overall decrease of dementia incidence, although sensitivity analyses showed that the intervention had a protective effect for non-Alzheimer dementia and a reduced occurrence of dementia in a subgroup of participants with baseline untreated hypertension [12].

A final consideration includes the ethical implications of advocating prevention to individuals who are healthy and most of whom would never develop dementia. This issue highlights the need for better predictive models to detect which individuals are most likely to progress to dementia, or who would benefit the most from the interventions, as well as the still unanswered questions about the long-term adherence to these lifestyle interventions. On the other hand, lifestyle interventions have minimal side effects and are beneficial to the individual regardless of their dementia risk.

### Conclusions and recommendations

“I only know that I know nothing”

Socrates

circa 470–399 BC

Our current understanding of the relationship between cognition and dementia pathology is incomplete with a lack of knowledge about temporality and sequence among lifestyle risk factors, appearance of disease-specific pathology, and the development of clinical symptoms. Nevertheless, the question “can dementia be prevented?” remains critical. Although brain β-amyloid accumulation is the most accepted hypothesis for the onset of AD, other concurrent mechanisms include tau accumulation, demyelination, neuroinflammation, metabolic dysfunction, and cerebrovascular changes. Moreover, the association between brain β-amyloid load and impeding dementia lessens with age, suggesting concurrent mechanism may be necessary for clinical expression [14]. Thus, if there are multiple pathways in the development of dementia, there may be multiple ways to treat, delay, or reduce the probability of developing it [15].

The conflicting points of view presented in this debate may pose a challenge as to what is best to recommend in practice for clinicians. Population attributable risk estimates show that age-specific incidence rates are declining in parallel with population-level reductions in risk factors such as low education, smoking, and cardiovascular disease, suggesting a potential public health effect when modifiable risk factors are treated. However, population attributable risk does not necessarily translate into recommendations for individuals. Conclusions drawn based on high-quality evidence from RCTs do not support the affirmative view and larger trials with longer follow-up may be needed. Open issues are the feasibility of conducting these large and long-term trials and how to untangle correlation from causation when treating risk factors. Mendelian randomization studies using genetic variants associated with the modifiable risk factors may shed light on causation; however, large samples are needed for these analyses.

The Lancet Commission on Dementia and the World Health Organization (WHO) Global Action Plan both acknowledge that dementia is a problem that mainly impacts low- and middle-income countries [1]. Due to the differential rates of population aging, the main global increase in dementia will occur in populous and rapidly aging middle-income countries like Brazil, China, and India. In low-income countries, lifestyle prevention treatments will be harder to implement since most are in the first stages of the WHO and Alzheimer’s Disease International’s six-stage model, where dementia is overlooked, the population lacks awareness, and the country does not have the necessary dementia infrastructure [16]. In addition, in low-income countries, fewer people are given the diagnosis of dementia or receive it when it is too late for them to benefit from lifestyle interventions [17]. Nevertheless, since even small reductions in dementia incidence would have a dramatic public health impact, one approach—based on observational evidence aforementioned in the affirmative view—is to recommend to patients to be physically and cognitively active, maintain healthy diets, and to manage their hearing loss and cardiovascular risk factors, particularly hypertension [18]. For example, there is robust observational evidence that diet is an important part of a healthy lifestyle, reducing the risk of developing cognitive impairment or dementia [19]. Evidence shows that the Mediterranean diet improves cognition in normal controls, although there is no support for cognitive impairment or dementia prevention yet, possibly because the effects require long-term dietary change [20]. Additionally no support for any positive effect when taking individual vitamin supplements [21].

Many of the lifestyle interventions proposed have health benefits beyond dementia and are relatively inexpensive with minimum adverse effects. Thus, even though evidence from clinical trials is less conclusive, recommendations for overall health could be made and add that they may also benefit brain health.

### Summary

Pharmacological treatments for dementia have not yielded the results that we had hoped for. Observational evidence suggests that it is possible to delay progression to dementia with lifestyle interventions targeting 9 risk factors, as noted in a reduction of age-related incidence of dementia in high-income countries. Conversely, evidence from large and high-quality RCTs using lifestyle interventions to modify some of these risk factors showed low effect sizes or no effect in improving cognition. Ultimately, since even relatively small reductions in dementia incidence would have a dramatic public health impact, one approach based on observational evidence is to encourage patients to be physically and cognitively active, maintain healthy diets, and to manage their hearing loss and cardiovascular risk factors, particularly hypertension. Current ongoing large lifestyle interventional trails, including those under the World Wide Finger initiative, may help to settle this debate [22, 23].

## Data Availability

Not applicable.
